# Machine learning approaches to drug response prediction: challenges and recent progress

**DOI:** 10.1038/s41698-020-0122-1

**Published:** 2020-06-15

**Authors:** George Adam, Ladislav Rampášek, Zhaleh Safikhani, Petr Smirnov, Benjamin Haibe-Kains, Anna Goldenberg

**Affiliations:** 1 Princess Margaret Cancer Centre, University Health Network, Toronto, ON Canada; 2 Department of Computer Science, University of Toronto, Toronto, ON Canada; 3 Vector Institute, Toronto, ON Canada; 4 Genetics and Genome Biology, Hospital for Sick Children, Toronto, ON Canada; 5 Department of Medical Biophysics, University of Toronto, Toronto, ON Canada; 6 Ontario Institute for Cancer Research, Toronto, ON Canada

**Keywords:** High-throughput screening, Combination drug therapy, Pharmacogenetics

## Abstract

Cancer is a leading cause of death worldwide. Identifying the best treatment using computational models to personalize drug response prediction holds great promise to improve patient’s chances of successful recovery. Unfortunately, the computational task of predicting drug response is very challenging, partially due to the limitations of the available data and partially due to algorithmic shortcomings. The recent advances in deep learning may open a new chapter in the search for computational drug response prediction models and ultimately result in more accurate tools for therapy response. This review provides an overview of the computational challenges and advances in drug response prediction, and focuses on comparing the machine learning techniques to be of utmost practical use for clinicians and machine learning non-experts. The incorporation of new data modalities such as single-cell profiling, along with techniques that rapidly find effective drug combinations will likely be instrumental in improving cancer care.

## Introduction

Cancer is a leading cause of death worldwide and the most important impediment to increasing life expectancy in every country of the world in the 21st century^1^. Fortunately, from 2011 to 2015, there has been a small but prominent decrease in death rates for all races/ethnicities combined for 11 out of 18 most common cancers among men and 14 of the 20 most common cancers among women. The continued decreases in death rates for colorectal cancer, prostate cancer and female breast cancer are largely due to advances in early detection and more effective treatments^2^. In this review, we will focus on the computational challenges of identifying the best treatment that improves chances of successful recovery.

Until recently, treatments were chosen based on the type of cancer in a one-size-fits-all manner. We are now witnessing the advent of precision oncology^3–5^ that takes into account patients’ genomic makeup for treatment decisions^3,6,7^. Treatment approval based on tumor-site agnostic molecular aberration biomarkers has become reality. The year 2017 marked the first FDA approval of such a treatment^8^. Based on clinical trials in 15 types of cancer, pembrolizumab was approved for treatment of solid tumors with mismatch repair deficiency or high microsatellite instability^9^. Larotrectinib is another promising treatment, targeting the tropomyosin receptor kinase gene fusion in a variety of cancers^10^. Unfortunately, there are no established biomarkers for majority of the anticancer drug compounds. Identification of reliable biomarkers is a challenge not only for the most commonly used cytotoxic drugs, but also in the case of targeted therapies as the drug targets alone are generally poor therapeutic indicators^11,12^.

**Fig. 1:**
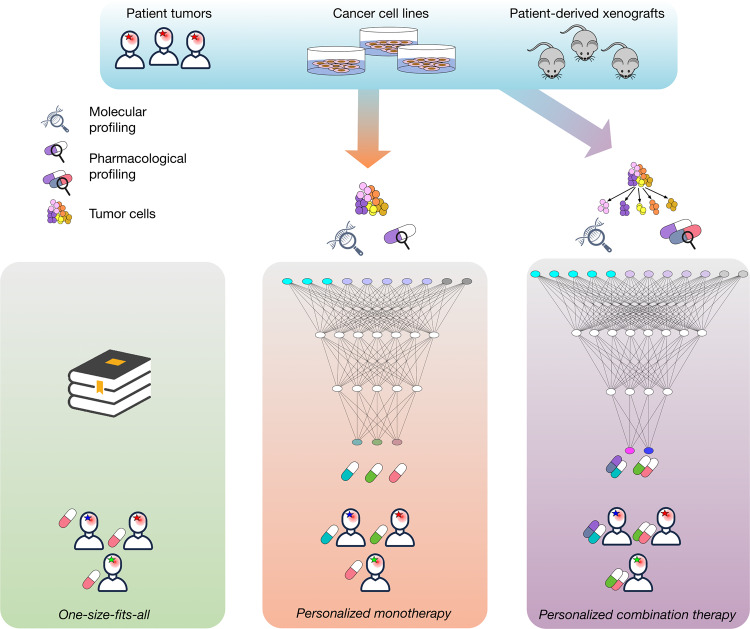
Graphical abstract. Patient data are limited, so to predict drug response, much of the existing literature use model system data, e.g. immortalized cell lines and PDX. **a** Currently most patients in cancer are still treated in a one-size-fits-all manner according to the type (or subtype) of cancer they have. **b** There is a growing number of examples of personalizing monotherapy in practice, where depending on the mutations in the tumor, the patient can be prescribed a targeted drug. This approach is applicable to fewer than 20% of the patients. The computational contribution is to take a large number of model systems and patients, when available and construct a predictive model to identify the best drug for majority of the patients. **c** Due to tumor heterogeneity and acquired drug resistance, monotherapies may not be effective, there is currently a growing body of work predicting drug synergy and effective drug combinations. Originally these models were trained using bulk data, but more recently, single-cell data-based approaches are starting to show promise. The person symbol in the figure was obtained from dryicons.com. The black magnifying glass is courtesy of Stanislav Tischenko under the Creative Commons Attribution 3.0 License.

Discovery of biomarkers predictive of drug response and development of multivariate companion diagnostics require efficient computational tools and substantial number of samples. Traditional statistical models and more sophisticated machine learning approaches have been used to build predictors of drug response and resistance both in the clinical^13^ and preclinical^14^ settings. As predictive models increase in complexity, the number of observations required to train these models increases as well. While omic profiles and clinical outcomes of patients are the most relevant data sources for the development of clinically relevant predictors, these datasets are often limited in size due to many factors including high costs, limited accrual rates, and complex regulatory landscape. In addition, by the nature of the experiment, unbiased testing of multiple therapeutic strategies for the same patient in the patient itself is practically infeasible. Cancer models provide access to patient tumors in preclinical models, both in vivo and in vitro, allowing researchers to test multiple drugs and combinations in parallel^14^. Although these preclinical models recapitulate patient therapy response to varying degrees, they provide massive amounts of pharmacogenomic data for drug response prediction. Here we review the recent applications of machine learning to prediction of response to monotherapies and identification of combination therapies (Fig. 1).

## Prediction of response to monotherapies

### In vitro and ex vivo tumor models

Large-scale efforts to associate molecular profiles with drug response phenotypes in preclinical models date back to the late 90s when the National Cancer Institute Developmental Therapeutics Program released large-scale pharmacogenomic data of 60 cancer cell lines (NCI60) screened with tens of thousands of chemical compounds, including a large panel of FDA-approved drugs^15^. NCI60 facilitated several drug discoveries, notably a 26S proteasome inhibitor bortezomib that is now used in multiple myeloma treatment^15^. Since then, high-throughput in vitro drug screens of cancer cell lines (CCLs) derived by immortalization of human cancer cells became popular experimental bases for discovery of multi-omic underpinnings of drug sensitivity and resistance^16^. Since this seminal study, multiple large-scale databases have been publicly released to the cancer research community^17,18^. More recently, advances in growing tumors in animal models enabled the generation of large collection of patient-derived xenografts (PDX) to monitor tumor growth with and without drug treatment in mice^19^. Novartis published the largest PDX-based pharmacogenomic dataset to date, referred to as the PDX Encyclopedia^20^. The NCI recently announced the Patient-Derived Models Repository (PDMR) with comprehensive molecular profiling and commitment to release pharmacological profiles in the future. A series of databases and tools have been developed recently to harmonize and make easily available multiple pharmacogenomic studies investigating anticancer monotherapies (Table 1).

**Table 1:** Platforms harmonizing preclinical pharmacogenomic datasets and providing basic processing functions for biomarker discovery.

Platforms	Cancer models	# Models	# Drugs	URL	References
PharmacoGx PharmacoDB	Cell lines	1691	759	https://bioconductor.org/packages/PharmacoGx/ http://pharmacodb.ca/	^17,111^
GDSCTools	Cell lines	1001	265	https://gdsctools.readthedocs.io	^112^
CellminerCDB	Cell lines	∼1000	~50,000	https://discover.nci.nih.gov/cellminercdb/	^113^
CancerDP	Cell lines	1061	24	http://crdd.osdd.net/raghava/cancerdp/index.php	^114^
PDXFinder	PDX	567	33	https://www.pdxfinder.org/	Unpublished
Xeva	PDX	277	61	https://github.com/bhklab/Xeva	^115^
Cancer-Drug eXplorer	2D cell cultures	462	60	http://cancerdrugexplorer.org/	^116^

### Methods for monotherapy prediction

The availability of commercial drug response prediction approaches is limited. In fact, publicly available methods mainly consist of biomarker assays which measure quantities such as gene expression and determine whether or not a specific therapy linked to the biomarker assay would be effective for a given patient. Most of these assays and predictive models are univariate, with only a few multivariate assays that are based on simple statistical and machine learning approaches (the OncotypeDx^21^ and MAMMAPRINT^22^ models for breast cancer are based on a linear regression model and a nearest centroid model, respectively). Thus, this review focuses on academic approaches to drug response prediction since they significantly outnumber commercial approaches, are more transparent, and address the more difficult task of predicting the efficacy of multiple drugs without knowing ahead of time the useful features for the task.

The most typical computational approaches to drug response prediction, specifically in preclinical models, consist of (1) quantification of drug response; (2) molecular feature selection or dimensionality reduction of the cellular measurements; (3) machine learning model fitting to predict drug response; and (4) model evaluation^23,24^. Multiple studies explored which genomic modalities harbor the most predictive signal of drug response by analyzing performance of predictive models. The most commonly utilized modalities include single nucleotide variations, copy number variations, RNA expression, methylation, and proteomics. Despite their widespread use in clinical settings, mutations and copy number variations have been shown to account for only a small subset of candidate biomarkers, while gene expression, methylation and protein abundance are regarded as the most predictive modalities^25–27^, each can be complemented by the multi-omic view of the cancer^28–30^. Perhaps the main obstacle in effectively leveraging all data modalities is fusing them while ignoring redundancies. A combined set of measurements can reach hundreds of thousands of features, while the number of available patients or cell lines remains in the hundreds. Such a high feature to sample ratio is bound to lead to overfitting where a model can perfectly fit the limited size training set, yet will have poor generalization performance when tested on new data. This limits the class of applicable predictive models to those with low complexity such as support vector machines or logistic regression since high complexity models like deep neural networks require many samples to avoid overfitting. Successful applications of deep learning in domains such as image classification or machine translation have worked due to a more favorable measurement to sample ratio (*N* > D) in addition to architectures that mimic the human brain and limit overfitting such as convolutional neural networks. Developing neural network architectures with an effective inductive bias for genomics will allow the complex underlying cancer biology to be better modeled compared to linear models which reduce risk of overfitting at the cost of introducing significant modeling bias. Another approach to deal with the limited number of samples typically available in drug response prediction experiments is feature selection. Feature selection removes features such as the gene expression of genes which are determined to be uninformative for the phenotype being predicted. This improves the ratio of features to samples, and a common to feature selection is univariate feature selection where only features highly corrected with the phenotype are kept. Multivariate approaches to feature also exist and consider sets of features at a time since any single feature individually might not be predictive of the outcome, but that does not imply that a collection of features is uninformative as well. Papillon-Cavanagh et al.^31^ identified univariate feature selection as a robust selection approach, later improved by minimum Redundancy, Maximum Relevance (mRMR) Ensemble feature selection^32^. Costello et al. and Jang et al. performed extensive comparative analyses of machine learning methods for drug response prediction in cancer cell lines, recommending using elastic net or ridge regression with input features from all genomic profiling platforms^27,29^. Costello et al. summarized a crowdsourced DREAM drug prediction challenge^29^, revealing two leading trends among the most successful methods. First, the importance of the ability to model nonlinear relationships between data and outcomes, and second, the incorporation of prior knowledge, e.g. biological pathways. The challenge winning model, Bayesian multitask multiple kernel learning method^33^, incorporated both of these approaches together with multi-drug learning^34^. Such multitask framing of the prediction problem is highly effective as it enables a more efficient use of available data when tuning parameters. Specifically, instead of building separate prediction models for each drug thereby using just a subset of the data, a single model trained with all the data that has some parameters shared amongst all the drugs, and some drug-specific parameters is the better choice.

Nonlinear relationships are of utmost importance since many cellular processes follow nonlinear dose-response relationships such as the activation of MAPK via Progesterone in oocytes^35^. Furthermore, models encoding prior biological knowledge have improved and more stable feature selection since noisy gene-level measurements can be abstracted into gene sets that have been experimentally validated to be involved in cancer-related processes. Lee et al.^36^ developed a method that integrates disease relevant multi-omic prior information to prioritize gene-drug associations. Most recently, Zhang et al.^37^ and Wang et al.^38^ introduced methods based on similarity network fusion and similarity-regularized matrix factorization, respectively, that take into account similarity among cell lines, drugs and targets. Drug chemical features and similarities were shown to be a promising additional information that can improve drug response prediction performance. There is no canonical way of incorporating drug features into most predictive models since it is difficult to encode how the drug features and omics features interact. Future models that address this shortcoming are likely to outperform competitors that do not, due to the highly informative content of molecular fingerprints. Specifically, a predictive model in a multitask setting can take compounds with known molecular targets, use the similarity computed between the molecular fingerprints, and more effectively tune parameters using similarity between compounds for parameter regularization.

### Deep learning methods for monotherapy prediction

The use of neural networks for drug response prediction dates back to the 90s. El-Deredy et al. showed that a neural network trained on tumor nuclear magnetic resonance (NMR) spectra data has potential as a drug response predictor in gliomas, and may be used to provide information about the metabolic pathways involved in drug response^39^. Neural networks, however, did not become a method of choice for monotherapy prediction yet. In fact, despite the recent prevalence of deep neural network (DNN) methods across many areas and industries, including related fields, such as computational chemistry^40–45^, DNNs have only fairly recently found their way into the drug response prediction. The reason for this is the typically low ratio of the number of samples to the number of measurements per sample that does not favor traditional feedforward neural architectures. Overparameterization in these models easily leads to overfitting and poor generalization to new datasets. However, in recent years, more public data has become available and newly developed deep neural network models are showing promise. For example, Chang et al.^46^ developed the CDRscan model, featuring a convolutional neural network architecture trained on a dataset of ~1000 drug response experiments per compound. Their model achieved significantly improved performance compared to other classical machine learning approaches such as Random Forests and SVM. Part of why CDRscan performed better than these baseline models resides in its ability to integrate genomic data and molecular fingerprints. In addition, its convolutional architecture has shown to be effective in many machine learning domains. Taking inspiration from already well-established neural architectures, and modifying their structure to properly handle genomic data is certainly a promising future direction.

Another promising direction is autoencoders that are able to learn from smaller datasets. An autoencoder is a neural network that compresses its input and tries to reconstruct the original data from the compressed representation. This is quite useful for feature extraction as shown by Way and Greene^47^ where a 5000 dimensional gene expression profile was compressed into just 100 dimensions, some of which represented phenotypically relevant features such as patient sex or melanoma status. Rampášek et al.^48^ evaluated semi-supervised variational autoencoders on monotherapy response prediction and developed an extension—a joint drug response prediction model, Dr.VAE, that leveraged pre- and post-treatment gene expression in cell lines, showing improved performance in drug response prediction on a variety of FDA-approved drugs compared comprehensively to many classical machine learning approaches. This improvement could potentially have been even greater if the model was setup in a multitask fashion in combination with molecular fingerprints. Dincer et al.^49^ developed DeepProfile, a method that combined variational autoencoders to learn 8-dimensional representation of gene expression in AML patients and then used this representation to fit a Lasso linear model for drug response prediction with improved performance compared to no feature extraction. Similarly, Chiu et al.^50^ pretrained autoencoders on mutation data and expression features on TCGA dataset and subsequently trained a deep drug response predictor. What differentiates their method from others is the use of pretraining. Pretraining allows for using unlabeled data from other sources such as TCGA, instead of just the gene expression profiles available from the drug response experiments, thereby significantly increasing the number of samples available and improving performance compared to using just the labeled data. The brief summary of methods is available in Table 2. The trend of model development shows that as more data become available and deep learning methods become better adapted to high dimensional/low sample size data, there is hope for convergence and creation of sophisticated models that will likely push the field of computational drug response prediction forward to eventually become clinically relevant.

**Table 2:** Computational tools for monotherapy prediction.

Name	Availability	Purpose	Methodology and features	Reference
HNMDRP	Matlab and R code	Drug response prediction in CCLs	Genomic and compound features combined with drug–target interaction and PPI	^37^ Source code: https://github.com/USTC-HIlab/HNMDRP
KRL	Python code	Drug prioritization (ranking) in CCLs transferable to patients	Kernelized rank learning using genomic features, (predominantly gene expression)	^117^ Source code: https://github.com/BorgwardtLab/Kernelized-Rank-Learning
CDRscan	Web Application^a^	Drug response prediction in CCLs	Deep neural network trained on somatic mutations and drug compound fingerprints	^46^
Dr.VAE	Python code	Drug response prediction in CCLs	Semi-supervised Variational Autoencoder of gene expression that incorporates drug perturbation effects	^48^ Source code: https://github.com/rampasek/DrVAE
CancerDP	Web Application	Drug response prediction in CCLs	SVM models using (combination of) genomic features (mutations, CNVs, expression levels)	^114^ Webserver: http://crdd.osdd.net/raghava/cancerdp/
BMTMKL	Matlab and R code	Drug response prediction in CCLs	Bayesian multiview (original genomic modalities + aggregated views) multitask model	^29^ Source code: https://github.com/mehmetgonen/bmtmkl

A non-exhaustive summary of the most recent monotherapy prediction methods with an available web service or source code.

^a^A web application has been promised by the authors, but no official implementation yet as of February 2020.

**Table 3:** Methods to infer tumor clonal composition from bulk DNA sequencing data.

Name	Using SSM or CNV for phylogeny reconstruction	Joint Deconvolution and Phylogeny inference?	Reference
PhyloWGS	SSM and percomuted CNV mixing proportion estimates	Joint Inference	^118^
Canopy	Both	Joint Inference	^119^
SPRUCE	SSM and percomuted CNV mixing proportion estimates	Joint Inference	^120^
PASTRI	SSM only	Two step clustering and Phylogeny Inference	^121^
PyClone	SSM only, corrects VAFs for CNV, does not use in reconstruction explicitly	Clustering and Identifying clonal genotypes only	^122^
SciClone	SSM only	Clustering and Identifying clonal genotypes only	^123^
THetA2	CNV only	Clustering and Identifying clonal genotypes only	^124^

## Resistance to monotherapy

While drug response prediction can help pick an optimal therapy given the current molecular characteristics of the cancer cells, tumors often exhibit drug resistance over the course of the treatment. Consequently, patients that respond initially to therapy regress as their cancer either adapts to overcome the chosen treatment, or an existing resistant subclone repopulates the tumor^51^. Understanding the common mechanisms cancers use to develop resistance can help inform treatment approaches to counteract this phenomena.

For therapies inhibiting the activity or signaling of their target, a common mechanism towards resistance is feedback selecting for upregulated expression of the target protein. For example, resistance to 5-FU has been demonstrated to arise from the amplification of its target thymidylate synthase (TS)^52^, with corresponding overproduction of TS enzyme and mRNA transcripts^53^. Furthermore, especially for tyrosine kinase inhibitors, tumors will evolve to re-activate pathways downstream of the targeted protein. A classical example is the resistance to the EGFR inhibitor Gefitinib which can often be explained by an acquired T790M mutation reducing drug binding affinity^54^.

For DNA damaging compounds or compounds inhibiting DNA repair, altered DNA damage response can lead to resistance. Studies have shown that treatment with cisplatin, a DNA damaging agent usually effective against BRCA deficient cancers, can lead to mutations restoring BRCA function and subsequently the activity of the Homologous Repair (HR) pathway^55,56^. Furthermore, studies suggest that secondary alterations to DNA damage response proteins can shift the response from the error-prone Non-Homologous End Joining pathway to HR, reducing sensitivity to DNA damaging agents^57^. Other mechanisms of resistance include modifications to enzymes involved in drug metabolism to either reduce conversion of drugs to active forms or deactivate the compound^58,59^, and more recently, intra-tumor heterogeneity (ITH)^60^. As this review is focuses on drug response prediction, not enough depth is provided to discussing how tumors acquire resistance to therapies, or how therapies work. Readers are referred to work by Holohan et al.^51^, Housman et al.^59^, and Malhotra and Perry^61^ for a comprehensive discussion on this topic. For more details on the biological complexity of cancer in general, readers are referred to the review articles by Blackadar^62^, and Bertram^63^.

## Combination therapies

Drug combinations are crucial for addressing the issue of drug resistance and preventing recurrence caused by a negligible amount of remaining cancer cells. Synergistic combinations can also reduce toxicity by allowing for lower doses of either drug to be used. By enabling reduced doses, drug combinations can further increase the feasibility of drug repurposing by increasing the potency of compounds that are only effective at clinically dangerous doses^64^.

Trial and error combination design has limited applicability in the clinic due to time constraints and potential hazardous exposure to toxic combinations without improving efficacy. For example, Hecht et al.^65^ performed a clinical trial for metastatic colorectal cancer (mCRC) patients involving the targeted compound bevacizumab, either oxaliplatin or irinotecan as a chemotherapeutic agent, and an optional addition of a human antibody panitumumab. The purpose of the trial was to evaluate benefit conferred by panitumumab. It was revealed that for the cohort that used oxaliplatin as a chemotherapeutic agent, survival was 5 months lower for patients that also received panitumumab, and there was a significant increase in adverse effects such as infections and pulmonary embolism compared to patients that did not receive panitumumab. Tol et al.^66^ also performed a clinical trial for mCRC, using combination of capecitabine, oxaliplatin, and bevacizumab, as the baseline treatment to investigate cetuximab. Patients that received cetuximab had a shorter progression-free survival and reported significantly more adverse effects compared to patients that did not receive cetuximab.

One promising direction for a setting where the goal is to study a constrained set of options to design an optimal treatment plan for a patient is adaptive trials via reinforcement learning^67^. The probabilistic ranking given by their method potentially allows for identifying when tumors develop drug resistance by analyzing when drug combinations are given priority over individual treatments. While this work, performed on PDX, learns more complex yet more effective policies in terms of survival than currently offered in the clinic, it is not clear how to mitigate the potential risks of exploration needed for reinforcement learning. We do hope that this direction is given its due consideration in the clinic since these early results appear to be very promising.

The limits of trial and error in the clinic can also be overcome in vitro with the use of preclinical models in the form of immortalized cancer cell lines or cell lines derived from patient biopsies. Patient-derived cancer models allow screening drug combinations in parallel without subjecting patients to serious toxicity risk (Table 4). Unfortunately, due to the sheer number of possible drug combinations, it is not possible to explore their potential antagonism, additive or synergistic effects^68^, so there is a need for methods that can predict combination therapy response prior to experimentally validating it.

**Table 4:** Drug combination datasets.

Dataset Name	Type	# Combinations	# Drugs	# Patients/cell Lines	URL	Ref
Drug Combination Database	Clinical	1363	904	~140,000	http://www.cls.zju.edu.cn/dcdb/	^125^
Merck	In vitro	583	38	39	http://mct.aacrjournals.org/highwire/filestream/53222/field_highwire_adjunct_files/3/156849_1_supp_1_w2lrww.xls	^126^
AstraZeneca-Sanger Drug Combination Dataset	In vitro	910	118	85	https://www.synapse.org/#!Synapse:syn4231880/wiki/235645	^30^
NCI ALMANAC	In vitro	5,000+	105	60	https://wiki.nci.nih.gov/display/NCIDTPdata/NCI-ALMANAC	^78^

### Methods for combination therapy prediction

Many computational methods have been developed to predict anticancer drug combination synergy based on a variety of genomic, drug structure, and biological network data. These methods vary in how much drug combination screening data is required, if used at all. Drug combination screening data refers to testing cancer models with combinations of two or more drugs rather than a single drug. A typical combination experiment setup involves testing two drugs at 8 different half-log dilution concentrations each including the null concentration as a control^69^. This gives rise to an 8×8 dose-response matrix. Using a 384-well assay plate, six pairs of drugs can be screened at once in this arrangement. Once cells are incubated in the wells for a sufficient amount of time, usually 72 h, a cell viability readout is conducted to determine the number of viable cells in each well. The collected data is then processed using a tool such as SynergyFinder^70^ to quantify the drug combination response compared to individual compound response based on a variety of models. As an example, the Bliss independence model^71^ provides a score under the assumption that the two drugs act independently, so measurement above this score indicates synergy. For more details on different synergy scores as well as experimental design of drug combination studies, the reader is referred to the experimental design guide by He et al.^69^. The number of experiments increases exponentially with the number of drugs tested in combination, making these combination screens both logistically complex and expensive. It is therefore favorable to have a method which does not require significant amounts of combination screens. Several approaches for drug synergy prediction described in the literature instead use a combination of either perturbation experiments or sensitivity experiments coupled with drug target and drug structure data. For example, the work done by Li et al.^72^ leverages gene expression perturbation data, measured as the difference in gene expression before and after treatment, to compute various statistics about differentially expressed genes as the main pharmacogenomic features. Additionally, the authors extracted drug physicochemical properties, distance between drug targets in PPI networks, and Jaccard similarity between targeted pathways to represent biological and chemical prior knowledge. These features were then used to train a random forest model to perform the binary prediction task of whether a drug combination is synergistic or not. Gayvert et al.^73^ also made predictions with random forests by using both single-drug response values and combination therapy response values when available. Interestingly, they did not leverage drug structure information nor gene expression profiles when making predictions. This is a drawback since drug structure information is easily available, and including it may improve performance, but it provides flexibility in not having to measure gene expression. However, their framework is broadly applicable, and their results indicate that even a small number of drug combination experiments can have a great performance benefit when used to train a model that makes predictions using primarily single-drug response data.

There is a class of drug combination optimization approaches that interacts with the user by suggesting promising combinations to test. Both Weiss et al.^74^ and Nowak-Sliwinska et al.^75^ use Feedback System Control (FSC) to iteratively refine drug combinations and suggest new ones to test in vitro. The process works by first starting with some randomly selected drug combinations for some range of doses. This group of combinations is then mutated using Differential Evolution (DE) to propose new drug combinations that are to be tested in vitro, and whose efficacy will be compared against the original randomly selected combinations. For each mutated combination, if that combination had higher efficacy than the original random combination that it was created from, then the new combination is kept, otherwise the original combination is kept. This procedure is repeated until some convergence criterion is met. This approach seems to be very effective in practice because the efficacy versus drug combination surface is smooth thereby allowing FSC to converge in 10–15 iterations. Lastly, the optimal drug combination identified by DE and evaluated in vitro is further optimized to eliminate redundant compounds or compounds having an antagonistic effects. Importantly, FSC based approaches are not limited in the number of drugs used in a given combination, unlike many methods that are created specifically for pairs of drugs. It might be possible to accelerate the convergence of FSC methods by including genomic or chemical data since both methods described above perform the optimization without considering drug targets or drug similarities.

### Deep learning methods for combination therapy prediction

The most extreme prediction scenario is to not use drug response data at all when building a model. This is done by Preuer et al.^76^ where the authors only leverage transcriptomic data and drug structure data to predict Loewe score which quantifies the excess over the expected response if the two drugs used in a combination were the same compound. What further differentiates this work from previous works is that the authors use deep learning to achieve state-of-the-art performance compared to baseline models such as gradient boosting machines, random forests, and support vector machines. Xia et al.^77^ used deep learning as a means of simultaneously extracting and integrating features from multiple data types to predict the efficacy of drug pairs. Combination response data as well as gene expression, microRNA, and protein abundance from the NCI-ALMANAC dataset was used^78^. Additionally, drug features were obtained using Dragon software^79^ which provides chemical fingerprints and other properties. Each data type was passed through its own submodel where a submodel is just a deep fully connected neural network in order to obtain useful features and perform dimensionality reduction. Then, these features for the different data types were concatenated and passed through a final submodel that uses residual connections in order to predict the drug combination score. Ultimately, the authors were able to obtain impressive results with R^2 of 0.92, and much of that explained variance was due to the drug descriptors. These approaches reinforce the importance of newer deep learning methods such as molecular graph convolution to extract task-specific molecular fingerprints. A summary of tools related to drug combinations is provided in Table 5. In terms of the availability, there are more synergy visualization tools rather than synergy prediction tools available to date. We hope that this trend will change as more researchers work on this important area and provide their tools in publicly available packages.

**Table 5:** Tools for visualizing, evaluating, and predicting synergistic drug combinations.

Name	Implementation	Purpose	Features	URL
SynergyFinder	Web Application	Evaluating Combo Efficacy	Has 4 different drug interactivity models Computes single-agent effects Computes synergy scores	https://synergyfinder.fimm.fi/
Combenefit	Desktop Application	Evaluating Combo Efficacy	Has 3 different drug interactivity models Meant to handle large batch experiments	https://www.cruk.cam.ac.uk/research-groups/jodrell-group/combenefit
CImbinator	Web Application	Evaluating Combo Efficacy	Has 1 drug interactivity model	http://cimbinator.bioinfo.cnio.es/CombinationIndex
DIGREM	Web Application	Evaluating Combo Efficacy	Models response curve and gene expression changes after treatment	http://lce.biohpc.swmed.edu/drugcombination/
RACS	R Package	In-Silico Synergy Prediction	Leverages drug target networks and transcriptomic profiles	https://github.com/DrugCombination/RACS
DeepSynergy	Web Application	Predicts Synergy Scores	Selects novel synergistic drug combinations	http://www.bioinf.jku.at/software/DeepSynergy/

## Drug combination discovery using single-cell sequencing

The development of single-cell sequencing technologies has given researchers a new set of tools to interrogate tumor heterogeneity. Single-cell DNA sequencing (scDNAseq), can be used to more directly investigate the clonal structure of a tumor. It works by isolating individual cells and performing whole genome amplification to increase the amount of DNA present in order to be detectable by a DNA sequencer^80^. These data can be used to directly reconstruct the unique genotypes as well as to estimate the clonal fraction within the sample. Bulk DNA sequencing does not have these abilities, so simply identifying populations of cells with different mutations can already significantly improve treatment plans (Table 3). However, scDNAseq data suffers from increased noise—each cell has only two copies of each genomic locus, requiring amplification before sequencing^81^. The amplification process can introduce errors into the sequenced reads, and amplification can be uneven across the genome as well as between cells, introducing bias into the observed reads. Computational approaches estimating tumor clonal composition while taking into account these sources of error have been developed^82–84^. For a thorough discussion of the methods used to analyze snDNAseq data, we refer the reader to the work by Qi et al.^85^. Interestingly, single-cell RNA sequencing (scRNAseq) is starting to be used to design novel drug combinations through identifying druggable subclones^86,87^. Unlike DNA, where each cell contains only one copy of each allele (to a total of 6 pg of DNA), there is approximately 30 pg of RNA in a single cell. With the advent of the Chromium platform it is also now possible to sequence the RNA across 100,000s of cells in a single experimental run^88^. Predictive models of drug response could be developed and trained using high-throughput preclinical pharmacogenomic data, and an optimization framework to predict the most efficient and the least toxic combination treatment could be established.

One of the first analyses to examine the influence of treatment on the transcriptome of cancer cells at single-cell resolution was conducted by Suzuki et al.^89^. They first performed single-cell sequencing on four different cell lines derived from lung adenocarcinoma to compare the relative divergence in their gene expression profiles. Even though the average gene expression levels were generally similar, the relative divergences between cell types were pronounced. To investigate how targeted therapy affects individual cells, they treated LC2/ad cell line and the derived resistant version of it with vandetanib, a multi-tyrosine kinase inhibitor. The comparison of single-cell profiles of treated cells versus parental cells identified a wide variety of genes overexpressed by drug stimulation. Particularly in case of LC2/ad, the diversity level of gene abundances between cells was significantly reduced after treatment so authors hypothesized that cells lose diversity in response to treatment. Interestingly, target genes of vandetanib, EGFR and RET, were not as affected by the treatment as some of the other off-target genes possibly due to the rigid transcriptional controls over these targets.

Kim et al.^90^ sequenced the transcriptome at single-cell resolution of a primary renal cell carcinoma (pRCC) and its lung metastasis (mRCC) from a patient and paired PDX models to design a combination therapy that would address the heterogeneous nature of the tumor. Whole exome sequencing of the metastatic sample and its PDX model indicated the preservation of major tumor features in the PDX model. In order to predict single-cell response of the RCC to the clinically approved drugs, activity of drug target pathways was estimated by conducting gene set enrichment analysis. Subsequently, cell lines derived from the PDX models were screened with the drugs. Predictive drug response models, based on ridge regression, were built using expression profiles of cancer cell lines from a publicly available drug screening dataset^91,92^ to predict response to the drugs. Authors used ComBat to remove the technical variation between the cell line dataset used for training, the drug response predictors, and single-cell RNA-seq data. Predicted drug response values were substantially correlated with measured sensitivity values (0.65). Accordingly, by considering high sensitivity prediction of cells to Afatinib and Dasatinib and mutually exclusive patterns in the activation status of their signaling pathways in cells, the authors suggested a combination of these two compounds as an efficient therapeutic strategy. In vitro validation in 2D and 3D cultured mRCC cells and in vivo validation in subcutaneous xenografts validated the expected additive effect of the drug combination over monotherapy responses. The administration of this combinatorial therapy is inducing superior growth inhibition by co-targeting mutually exclusive EGFR and Src signaling pathways.

One of the major weaknesses of Kim et al.’s^90^ work is the low number of single cells sequenced. The captured cells may not reflect the true clonality of the patient tumor and might even lead to false discoveries. Recent technological advances in single-cell sequencing made it feasible to capture large numbers of single cells in one experiment. New computational pipelines and approaches have been developed to improve all the steps in processing of the single-cell sequencing data^93,94^, including tackling noise and dropout in these experiments, normalization techniques, dimensionality reduction^95–97^ and clustering approaches^98,99^. These rapidly evolving methodologies provide remarkable opportunities for the discovery of biomarkers, prediction of efficient therapies, and the study of mechanisms of acquiring resistance to treatments.

Anchang et al.^100^ were the first to use single-cell perturbation experiments to optimize drug combinations. Their model DRUG-NEM required the specification of lineage, intracellular communication, and apoptosis markers that were measured in drug perturbation experiments using Mass Cytometry Time-of-Flight (CyTOF). The objective of the model is to select the minimum number of drugs that creates the maximum perturbation effect on the markers of interest using perturbation data from single-drug experiments. Drug effects were measured using a Bayesian linear model to compute the probability that an intracellular communication marker is differentially expressed between treatment and control. A graphical model is then created from these probabilities using a nested effects model, and all the possible drug combinations are ranked. This approach is limited by having to know ahead of time which markers to use, and this in turn requires knowing the mechanisms of action for the drugs, which in many cases is not available. Nevertheless, this direction for drug response prediction is very promising and will be greatly aided by the burgeoning single cell and drug clonality research.

## Opportunities and challenges: data and deep learning

The only standardized metric to date for cancer response is RECIST, and it relies on imaging data, mainly CT and MRI, to determine how tumors grow or shrink in patients. RECIST can handle up to 10 lesions in the patient, prioritized based on the largest lesions, and uses the sum of the lesion diameters (LD) when first measured as the baseline value. In subsequent scans, response is categorized into 4 different categories based on how much the sum of LDs has changed: complete response, partial response, stable disease, and progressive disease. There is no such international standard used to measure response for in vitro preclinical models and RECIST is usually not used in in vivo preclinical models due to costs, thus prohibiting fair comparisons between response prediction methods. Furthermore, some drug response prediction studies frame the task as regression where continuous values such as IC50 are predicted, and others frame the task as classification where a binary value which indicates inhibition or growth is predicted. Reproducibility between cell line based drug response studies remains a challenge due to differences in viability assays, drug concentrations, and cell seeding density^101^. There is also a need for better data sharing as technical replicates are necessary for estimating within-study variability, yet are sometimes not publicly released^102^. Additionally, the studies use a variety of datasets, thereby making quantitative comparisons even less feasible. Instead, qualitative comparisons are made between the methods that consider data requirements, generalization ability, and capacity to model complex biological interactions and chemical interactions. These comparisons are of great practical use as they provide context and scenarios in which one method is likely better than another.

The success of deep learning across scientific fields followed the collection of large standardized datasets. An additional factor important to broad utilization of deep learning was the growth in available computational power for training these models. Similarly, successful applications of deep learning in predictive oncology followed the growth of high-throughput preclinical datasets. This suggests that with additional data from studies that are more reproducible, deep learning could provide significant improvements over traditional machine learning methods in drug response prediction and drug combination prioritization. Specifically, the end-to-end nature of deep learning allows for extremely effective feature extraction and also enables the integration of multiple distinct data modalities. Additionally, encoding prior biological knowledge in neural networks can be done via several mechanisms such as graph-convolution networks^103^, or conditional scaling which allows for multiplicative relations between features such as a mutation being required for gene expression levels to be relevant. The nonlinear nature of deep neural networks, combined with their inductive bias that allows them to generalize even though they have many more parameters than samples, suggests that promising applications are possible in pharmacogenomics where complex correlation structures exist among features and between features and labels. For example, graph convolutional networks are a promising new way of encoding structural information from molecular graphs^104^ and can give application-specific chemical fingerprints that are more specialized for drug response or combination therapy discovery. Another fruitful direction is the use of transfer learning to leverage an abundance of omics data already available. The main obstacle for transfer learning is the large discrepancies between the techniques and experimental protocols used for different studies which lead to batch effects that violate the assumptions on which deep learning relies to generalize to new datasets. The creation of domain adaptation techniques, similar to computer vision^105^, specific for omics data will be of immense help in enabling transfer learning. Still, creating architectures with an effective inductive bias for processing omics data is difficult since it is not possible to just rely on the human brain for inspiration like in image analysis. Thus, neural architecture search techniques which remove humans from the design loop by automating the creation and testing of architectures are of key importance in making deep learning more successful in drug response prediction^106^. It has recently been shown that the success of architecture search techniques depends significantly on careful design of the search space^107^. This requires encoding prior knowledge about potentially effective architecture choices which is certainly less difficult than specifying an entire architecture, but still remains challenging. Deep learning can certainly help in better understanding cancer biology by predicting binding sites or discovering new biomarkers by analyzing RNA transcripts^47,108,109^. In fact, deep learning has also been used to predict protein-protein interactions^109^ which are of increasing interest as potential targets for cancer therapies^110^, so deep learning will have an impact on both drug discovery and drug response prediction.

The problem of predicting the optimal treatment or combination of treatments for a cancer patient remains unsolved. The approaches reviewed above seek to bring recent advances in machine learning to bear on this challenge, leveraging the growing high-throughput preclinical screening data and new technologies allowing the profiling of tumors on a single-cell level. Promising results in this area should encourage both the investigators working on developing cheaper and more precise high-throughput screens to enable further data collection as well as ML method developers to develop novel tools incorporating peculiarities of cancer biology. While there remains much work to be done, the field is nascent and offers a path to a truly personalized approach to oncology.

## Supplementary information

Supplementary Material
