# Supplementary material for: Machine learning approaches to drug response prediction: challenges and recent progress
Source: NPJ Precis Oncol. 2020 Jun 15;4:19. doi: 10.1038/s41698-020-0122-1 (PMC7296033; doi:10.1038/s41698-020-0122-1)
Supplement: Supplementary file 1 — Supplementary Material [file 41698_2020_122_MOESM1_ESM.pdf]

Supplementary Table S1. List of publicly available clinical trial studies.

Study	# Patients	Tissues	Molecular profiling	Drugs	Source
NEOALTTO	89	Breast (HER2+)	WXS; RNA-seq	Lapatinib	NCT00553358
NEOALTTO	79	Breast (HER2+)	WXS; RNA-seq	Trastuzumab	NCT00553358
NEOALTTO	86	Breast (HER2+)	WXS; RNA-seq	Lapatinib + Trastuzumab	NCT00553358
Bortezomib_Myeloma	169	Purified myeloma	microarray (Affymetrix 133A/B)	bortezomib	https://www.ncbi.nlm.nih.gov/geo/query/acc.cgi?acc=GSE9782
Epirubicin_ER-	118	Breast (ER-)	HG-U133_Plus_2	epirubicin	https://www.ncbi.nlm.nih.gov/geo/query/acc.cgi?acc=GSE16446
Breast_Neoadj	506	Breast	HG-U133A	neoadjuvant taxane-anthracycline chemotherapy	https://www.ncbi.nlm.nih.gov/geo/query/acc.cgi?acc=GSE25066
TransNOAH	Total 156	Breast (HER2+)	HG-U133_Plus_2	trastuzumab	https://www.ncbi.nlm.nih.gov/geo/query/acc.cgi?acc=GSE50948
TransNOAH	Total 156	Breast (HER2+)	HG-U133_Plus_2	trastuzumab+neoadjuvant chemo	https://www.ncbi.nlm.nih.gov/geo/query/acc.cgi?acc=GSE50948
Breast_Neoadj2	115	Breast	HG-U133_Plus_2	neoadjuvant paclitaxel followed by 5-fluorouracil/epirubicin/cyclophosphamide	https://www.ncbi.nlm.nih.gov/geo/query/acc.cgi?acc=GSE32646
Breast_Neoadj3	279	Breast	HG-U133A	Neoadjuvant chemotherapy	https://www.ncbi.nlm.nih.gov/geo/query/acc.cgi?acc=GSE41998
Breast_Chemo	69	Breast (TNBC)	HG-U133A	chemotherapy	https://www.ncbi.nlm.nih.gov/geo/query/acc.cgi?acc=GSE58479
Breast_Neoadj5	278	Breast	HG-U133A	Neoadjuvant chemotherapy	https://www.ncbi.nlm.nih.gov/geo/query/acc.cgi?acc=GSE20194
Breast_Chemo2	Total 161	Breast	U133_X3P	(5-fluorouracil, cyclophosphamide, epirubicin)	https://www.ncbi.nlm.nih.gov/geo/query/acc.cgi?acc=GSE6861
Breast_Chemo2	Total 161	Breast	U133_X3P	(epirubicin, docetaxel)	https://www.ncbi.nlm.nih.gov/geo/query/acc.cgi?acc=G

					SE6861
Breast_Chemo3	178	Breast	HG-U133A	chemotherapy	https://www.ncbi.nlm.nih.gov/geo/query/acc.cgi?acc=GSE20271
Breast_Gefitinib	Total 96	Breast	HG-U133_Plus_2	Neoadjuvant anastrozole	https://www.ncbi.nlm.nih.gov/geo/query/acc.cgi?acc=GSE48906
Breast_Gefitinib	Total 96	Breast	HG-U133_Plus_2	Neoadjuvant anastrozole+Gefitinib	https://www.ncbi.nlm.nih.gov/geo/query/acc.cgi?acc=GSE48906
Breast_Letrozole	58	Breast	HG-U133A (before and after treatment)	letrozole	https://www.ncbi.nlm.nih.gov/geo/query/acc.cgi?acc=GSE20181
Breast_Chemo4	52	Breast	Expression array	gemcitabine, epirubicine and docetaxel	https://www.ncbi.nlm.nih.gov/geo/query/acc.cgi?acc=GSE4056
